# Prevalence of celiac disease in Iranian patients with rheumatologic disorders 

**Published:** 2018

**Authors:** Elham Elhami, Zahra Zakeri, Amir Sadeghi, Mohammad Rostami-Nejad, Umberto Volta, Mohammad Reza Zali

**Affiliations:** 1 *Labafinejad Hospital, Shahid Beheshti University of Medical Sciences, Tehran, Iran*; 2 *Basic and Molecular Epidemiology of Gastrointestinal Disorders Research Center, Research institute for Gastroenterology and Liver Diseases, Shahid Beheshti University of Medical Sciences, Tehran, Iran *; 3 *Gastroenterology and Liver Diseases Research Center, Research Institute for Gastroenterology and Liver Diseases, Shahid Beheshti University of Medical Sciences, Tehran, Iran*; 4 *Department of Medical and Surgical Sciences, University of Bologna, Italy *

**Keywords:** Celiac Disease, Fibromyalgia, Rheumatoid Arthritis, Systemic Lupus Erythematosus

## Abstract

**Aim:**

Patients with Systemic Lupus Erythematosus (SLE), Rheumatoid Arthritis (RA), and Fibromyalgia (FM) may have underlying non-diagnosed celiac disease (CD).

**Background:**

The aim of this study was to determine the prevalence of CD in patients with these underlying diseases in Iran.

**Methods:**

This cross-sectional study was performed among 300 consecutive patients with SLE, RA, and FM (each group 100 patients) since 2015 to 2017. The blood samples were collected and serum IgA anti-tissue trans-glutaminase (Anti-tTG) level was assessed for all patients. The seropositive patients underwent endoscopy and duodenal/jejunal biopsy according to the Marsh classification.

**Results:**

Out of 300 investigated patients with mean age of 41.2 years old, 92% of patients with SLE, RA and fibromyalgia were women. Among 100 patients with SLE, only 1 subject (1%), out of 100 patients with RA 3 subjects (3%), and none of the patients with fibromyalgia were seropositive for CD (with overall prevalence 1.4). All four patients were female and categorized as Marsh III.

**Conclusion:**

The results of the study indicated that patients with lupus have the same prevalence, but subjects with RA had three times higher prevalence rate than normal population for CD. Therefore, CD investigation in these individuals can improve their quality of life.

## Introduction

 Celiac disease (CD) is a lifetime gluten-induced autoimmune disease of small intestine affecting vulnerable individuals with worldwide and local prevalence rate of one and three percent, respectively. The diagnosis is based on serological antibodies and positive biopsy upon gluten-containing diet (1). The role of gastrointestinal factors in rheumatologic diseases, was firstly described by Smith in early 1920s in patients with reactive arthritis, ankylosing spondylitis, and rheumatoid arthritis (2,3). Inconsistent studies reported the association between CD and Systemic Lupus Erythematosus (SLE), Rheumatoid Arthritis (RA), and Fibromyalgia (FM) (4-13).

According to the previous studies patients with CD would have three times higher rate of rheumatologic diseases and follow-up in untreated celiac patients may lead to diagnosis of them (4, 5). Multi-factorial status such as environmental and genetic predisposition may be the cause of association between rheumatologic diseases and CD especially in relation with Toll-Like Receptors (TLR) and multiple cytokines and chemokines as a part of autoimmune spectrum (14-17). There are multiple HLA and non HLA genes which are the same for celiac and rheumatologic diseases (17). Some incomplete atypical rheumatologic diseases forms are related to Gliadin activity (18). Among the other etiological factors for association of CD and SLE, ultraviolet exposure and hypovitaminosis-D are common predisposing factors (19-25). Few studies have previously investigated the association between RA and CD in Iran, but we did not find any documents regarding this association with SLE and FM in the literature. Therefore, the aim of this study was to assess the prevalence of celiac in patients with SLE, RA, and FM in Iranian population. 

## Methods

In this cross-sectional study, 300 patients with SLE, RA, and FM (each group compromise 100 patients) referred to Labbafinejad Hospital from 2015 to 2017 were enrolled. Inclusion criteria were presence of SLE, RA or FM and exclusion criteria were known history of gastrointestinal disease or surgery. Diagnosis criteria were made by rheumatologist according to ACR criteria. A patient satisfied diagnostic criteria for FM if the following 3 conditions were met; widespread pain index; symptoms had been present at a similar level for at least 3 months; the patient had no disorder that would otherwise explain the pain. 

The criteria for SLE are mentioned in the table 1.

Target population for RA (Who should be tested?): Patients: 

1. Who have at least 1 joint with definite clinical synovitis (swelling)*

*2. With the synovitis not better explained by another disease†*

*Classification criteria for RA (score-based algorithm: add score of categories A–D;*

*a score of _6/10 is needed for classification of a patient as having definite RA) ‡*

*A. Joint involvement§*

*1 large joint. 0*

*2_10 large joints 1*

*1_3 small joints (with or without involvement of large joints) # 2*

*4_10 small joints (with or without involvement of large joints) 3*

5_10 joints (at least 1 small joint) ** 5

*B. Serology (at least 1 test result is needed for classification) ††*

*Negative RF and negative ACPA 0*

*Low-positive RF or low-positive ACPA 2*

*High-positive RF or high-positive ACPA 3*

*C. Acute-phase reactants (at least 1 test result is needed for classification) ‡‡*

*Normal CRP and normal ESR 0*

*Abnormal CRP or abnormal ESR 1*

*D. Duration of symptoms§§*

*Less than 6 weeks 0*

*More than 6 weeks 1*

All patients were assessed by clinical examination and medical history taking. 5 ml blood samples were collected and the level of Anti-tTG IgA (PharmaChimi, Iran) and serum IgA (Parsazmon, Iran) were assessed by ELISA kit and Immunoturbidimetry, respectively, according the manufacture instructions. Patients with IgA deficiency in the population studied were excluded using Anti-tTG IgG. The informed consent form was attained from all participating patients for blood sampling and Helsinki Declaration was respected all over the study. The study was approved by ethic committee of Research institute for Gastroenterology and Liver Diseases, Shahid Beheshti University of Medical Sciences, Tehran, Iran.

A seropositive patient who had tTG-IgA more than 10 IU/ml in ELISA test underwent endoscopy and also duodenal/jejunal biopsy (3 to 4 samples) for histological assessment according to the Marsh classification. The patients showing villous atrophy consistent with CD diagnosis underwent gluten-free regimen and after six months were rechecked for therapeutic response. Data were collected by checklist including demographic, clinical, and laboratory results.

Data analysis was performed by SPSS (version 20.0) software [Statistical Procedures for Social Sciences; Chicago, Illinois, USA]. Chi-Square, Fisher, and ANOVA tests were used and were considered statistically significant at P values less than 0.05. 

**Table 1:** Diagnostic criteria for systemic lupus erythematosus

Criterion for SLE	Definition
1. Malar rash	Fixed erythema, flat or raised, over the malar eminences, tending to spare the nasolabial folds
2. Discoid rash	Erythematous raised patches with adherent keratotic scaling and follicular plugging; atrophic scarring may occur in older lesions
3. Photosensitivity	Skin rash as a result of unusual reaction to sunlight, by patient history or physician observation
4. Oral ulcers	Oral or nasopharyngeal ulceration, usually painless, observed by physician
5. Nonerosive arthritis	Involving 2 or more peripheral joints, characterized by tenderness, swelling or effusion
6. Pleuritis or pericarditis	1. Pleuritis -- convincing history of pleuritic chest pain or rubbing heard by a physician or evidence of pleural effusion OR 2. Pericarditis -- documented by electrocardiogram or rub or evidence of pericardial effusion
7. Renal disorder	1. Persistent proteinuria > 0.5 g/day or > than 3+ if quantitation not performed OR 2. Cellular casts -- may be red cell, hemoglobin, granular, tubular, or mixed
8. Neurologic disorder	1. Seizures OR 2. Psychosis (Both in the absence of offending drugs, or known metabolic derangements, eg, uremia, ketoacidosis, or electrolyte imbalance)
9. Hematologic disorder	1. Hemolytic anemia -- with reticulocytosis OR 2. Leukopenia -- < 4000/mm3 on > 2 occasions OR 3. Lymphopenia -- < 1500/mm3 on > 2 occasions OR 4. Thrombocytopenia -- <100,000/mm3 in the absence of offending drugs
10. Immunologic disorder	1. Anti-DNA antibody to native DNA in abnormal titer OR 2. Anti-Sm presence of antibody to Sm nuclear antigen OR 3. Positive finding of antiphospholipid antibodies on: a. Abnormal serum level of IgG or IgM cardiolipin antibodies b. Positive test for lupus anticoagulant using a standard method or c. False-positive test for at least 6 months confirmed by *Treponema pallidum *immobilization or fluorescent treponemal antibody absorption test
11. Positive antinuclear antibody	An abnormal titer of antinuclear antibody by immunofluorescence or an equivalent assay at any point in time and in the absence of drugs

**Table 2:** Frequency distribution of clinical symptoms across the groups

Sign/ Symptom / History	SLE n (%)	FM n(%)	RA n(%)	CD (+) (n=4)		
				SLE n(%)	FM n(%)	RA n(%)
Infertility	1 (1)	0	0	0	0	0
Type I DM	2(2)	1(1)	1(1)	0	0	0
History of Abortion	2(2)	1(1)	1(1)	0	0	0
History of Smoking	2(2)	2(2)	1(1)	0	0	0
Diarrhea	5 (5)	10(10)	6(6)	0	0	0
Dyspepsia	15(15)	12(12)	9(9)	0	0	0
Belching	5(5)	8(8)	4(4)	1(34)	0	2(78)
Bloating	10(10)	3(3)	7(7)	0	0	0
Gas Passing	17(17)	11(11)	13(13)	0	0	0
Constipation	6(6)	14(14)	11(11)	0	0	0
Abdominal Pain	5(5)	27(27)	18(18)	0	0	0
GERD	5(5)	36(36)	14(14)	1(34)	0	2(67%)
Nausea/Vomiting	22(22)	26(26)	29(29)	0	0	0
Fatigue	2(2)	11(11)	9(9)	0	0	0
Musculoskeletal Pain	39(39)	63(63)	37 (37)	1(34)	0	3(100)
Depression	9(9)	53(53)	16(16)	0	0	0
Anxiety	14(14)	44(44)	19(19)	1(34)	0	1(34)

## Results

Of the 300 patients with lupus, rheumatoid arthritis and fibromyalgia, 276 (92%) were female and 24 (8%) were male. Of 100 patients with lupus with mean age 44.9 ± 5.7 (range 41 to 60), 92 were female (92%) and 8 (8%) were male. Of 100 patients with RA with mean age 38.8 ± 5.3 (range 32 to 47), 90 women (90%) and 10 patients (10%) were male. Of 100 patients with FM with mean age 42.2 ± 5.5 (range 33 to 51), 94 women (94%) and 6 patients (6%) were male.

Among patients with SLE, only 1 subject (1%), among RA patients, 3 subjects (3%), and none of the patients with FM were seropositive for CD (with overall prevalence 1.4). IgA deficiency was not reported in any cases. All seropositive cases underwent endoscopy and histopathological evaluation confirmed CD diagnosis in all of them. The histological examinations showed that all CD patients were female and categorized as Marsh III. After 6 months GFD, the serological marker was backed to normal in all CD patients.

The distribution of clinical symptoms across the groups is shown in table 2. Musculoskeletal pain was the most predominant symptom in all studied groups (SLE: 39%; FM: 63% and RA: 37%). Also nausea/vomiting 22(22%) and gas passing 17(17%) in SLE patients, depression 53(53%) and GERD 36(36%) in FM and nausea/vomiting 29(29%) and anxiety 19(19%) in RA was the more frequent symptoms. The common symptoms in CD patients was musculoskeletal pain and belching.

## Discussion

In current study the prevalence of CD in patients with SLE, RA, and FM was assessed and the result showed that the frequency of CD in Iranian patients with rheumatologic disorders is 1.4% (four CD patients including three with RA and one with SLE). In a review study in Iran, Rostami Nejad *et al.* (25) reported that the prevalence of this disease in general population in different parts of the country is between 0.5 and 1 percent which is similar to European and United States studies (17, 18). 

Nutritional factors may have an effect on the relationship between CD and SLE. Studies have shown that people with CD are at higher risk for SLE than the general population, but the absolute risk is low (4). Freeman (4) reported that among 264 celiac patients, six (2.4%) had SLE in follow-up. On the other hands, Catassi *et al.* (23) showed that all 24 seropositive out of 103 SLE patients were negative in pathological assessment for CD. Shani *et al*. (6) reported that CD was significantly more common in SLE patients with a rate of 0.8% versus 0.2% compare with healthy subjects. Similar to Shani *et al*. study, we found that the prevalence of CD in SLE patients was 1% (CI95%: 0.1 to 1.2). Ben-Abdelghani *et al.* (24) found that among 24 SLE patients, 8% had positive anti-tTG antibody but only one patient was approved by pathological assessment. However, according to Freeman study longer follow-up would results in higher prevalence rate (4). 

Gluten-free diet had a direct impact on patients’ quality of life with FM syndrome that had CD at the same time. Rodrigo *et al.* (8, 10) reported 6.7% rate for CD in FM patients who all were Marsh III and had therapeutic response to gluten-free regimen. In the other hand, in accordance to our finding, Tovoli *et al.* (9) reported no association between FM and CD.

The previous studies indicated that the possible causes of CD in RA are HLA predisposing role, immunological etiologies, family-clustering, geographic factor effects, and nutritional predisposition. In contrast with our findings, Moghtaderi *et al*. (12) did not find any cases of CD among patients with RA. James *et al*. (28) reported only one case of CD among 160 RA patients with pathology confirmation. Differently for our results, Coaccioli *et al*. (13) reported no celiac case in RA patients versus 0.5 to 1 percent in general population (29). The prevalence rate of CD in RA patients in our study was 3% (CI 95% 0.2 to 3.2) which is 3 times higher than general population. This prevalence is quite higher than previously reported (13, 28). In line with Rodrigo *et al*. (8) all our patients were in Marsh III stage. 

In conclusion, the results of the study indicated that patients with SLE have the same prevalence, but patients with RA have three times higher prevalence rate than normal population for CD. Therefore, the investigation of larger sample size with follow-up would be beneficial to attain more definite results and can improve the patient’s quality of life.

## Conflict of interests

The authors declare that they have no conflict of interest.
